# Symptom‐based staging for logopenic variant primary progressive aphasia

**DOI:** 10.1111/ene.16304

**Published:** 2024-04-26

**Authors:** Chris J. D. Hardy, Cathleen Taylor‐Rubin, Beatrice Taylor, Emma Harding, Aida Suarez Gonzalez, Jessica Jiang, Laura Thompson, Rachel Kingma, Anthipa Chokesuwattanaskul, Ffion Walker, Suzie Barker, Emilie Brotherhood, Claire Waddington, Olivia Wood, Nikki Zimmermann, Nuriye Kupeli, Keir X. X. Yong, Paul M. Camic, Joshua Stott, Charles R. Marshall, Neil P. Oxtoby, Jonathan D. Rohrer, Frankie O'Shea, Anna Volkmer, Sebastian J. Crutch, Jason D. Warren

**Affiliations:** ^1^ Dementia Research Centre, UCL Queen Square Institute of Neurology UCL London UK; ^2^ Uniting War Memorial Hospital Sydney New South Wales Australia; ^3^ Faculty of Medicine, Health and Human Sciences Macquarie University Sydney New South Wales Australia; ^4^ UCL Centre for Medical Image Computing, Department of Computer Science University College London London UK; ^5^ Division of Neurology, Department of Internal Medicine, King Chulalongkorn Memorial Hospital Thai Red Cross Society Bangkok Thailand; ^6^ Cognitive Clinical and Computational Neuroscience Research Unit, Faculty of Medicine Chulalongkorn University Bangkok Thailand; ^7^ HealthAbility Melbourne Victoria Australia; ^8^ Division of Psychiatry, Marie Curie Palliative Care Research Department UCL London UK; ^9^ ADAPTlab, Research Department of Clinical, Educational and Health Psychology UCL London UK; ^10^ Centre for Preventive Neurology Queen Mary University of London London UK; ^11^ Psychology and Language Sciences (PALS) UCL London UK

**Keywords:** Alzheimer's disease, logopenic, primary progressive aphasia, staging

## Abstract

**Background and purpose:**

Logopenic variant primary progressive aphasia (lvPPA) is a major variant presentation of Alzheimer's disease (AD) that signals the importance of communication dysfunction across AD phenotypes. A clinical staging system is lacking for the evolution of AD‐associated communication difficulties that could guide diagnosis and care planning. Our aim was to create a symptom‐based staging scheme for lvPPA, identifying functional milestones relevant to the broader AD spectrum.

**Methods:**

An international lvPPA caregiver cohort was surveyed on symptom development under an ‘exploratory’ survey (34 UK caregivers). Feedback from this survey informed the development of a ‘consolidation’ survey (27 UK, 10 Australian caregivers) in which caregivers were presented with six provisional clinical stages and feedback was analysed using a mixed‐methods approach.

**Results:**

Six clinical stages were endorsed. Early symptoms included word‐finding difficulty, with loss of message comprehension and speech intelligibility signalling later‐stage progression. Additionally, problems with hearing in noise, memory and route‐finding were prominent early non‐verbal symptoms. ‘Milestone’ symptoms were identified that anticipate daily‐life functional transitions and care needs.

**Conclusions:**

This work introduces a new symptom‐based staging scheme for lvPPA, and highlights milestone symptoms that could inform future clinical scales for anticipating and managing communication dysfunction across the AD spectrum.

## INTRODUCTION

Communication difficulties are a significant clinical issue across the phenotypic spectrum of Alzheimer's disease (AD) [1, 2, 3, 4, 5] but most salient in its language‐led variant, logopenic primary progressive aphasia (lvPPA) [4, 5, 6]. Language and communication functions are not comprehensively captured by standard clinical rating scales developed for typical AD [7]. It has recently been shown that symptom‐based staging informed by lived experience is feasible for other primary progressive aphasia (PPA) syndromes [8]; however, a similar tool to signpost the evolution of communication problems is lacking for lvPPA. This is particularly urgent with the advent of disease‐modifying therapies for AD, as the eligibility of individuals with lvPPA and other forms of ‘atypical’ AD remains unclear [9] and may be confounded by misleadingly poor performance on standard, language‐weighted cognitive tests.

Here a new, clinical, symptom‐based staging scheme for lvPPA is presented, informed by surveyed caregivers and emphasizing cognitive and functional ‘milestones’ of illness onset and progression.

## METHODS

### Exploratory survey

Following previously described methods [8], two of the authors (CJDH and JDW) suggested an initial list and sequence of symptoms associated with lvPPA, based on (i) clinical observations within the PPA cohort at the Dementia Research Centre; (ii) thorough examination of case notes for patients with lvPPA seen in the Specialist Cognitive Disorders Clinic at the National Hospital for Neurology and Neurosurgery, University College London; and (iii) a narrative review of the published literature on lvPPA, as summarized in the Introduction. Symptoms encompassed aspects of verbal communication (A) and non‐verbal functioning (including non‐verbal thinking and personality, B1; and personal care and wellbeing, B2). Qualitative input on these symptoms was gathered from members of the UK national PPA Support Group [10], via an online survey hosted on the Opinio platform. Data collection took place between October 2018 and January 2019, and respondents comprised 34 caregivers of individuals with lvPPA, all of whom had longstanding personal contact with the patients whose illness they described. Using findings from this exploratory survey, the list of symptoms was broadened and a preliminary six‐stage framework was devised for ordering functional impairment symptoms specific to lvPPA, ranging from stage 1 (least severe) to stage 6 (most severe). To assist caregivers with their responses, descriptions of the daily‐life consequences for each stage were included. These descriptions were derived from the Reisberg Global Deterioration Scale [11] and descriptors used previously in stages for two other rare dementias: posterior cortical atrophy and frontotemporal dementia [12, 13] (see Table S1).

### Consolidation survey

These provisional stages were next entered into another online, mixed‐methods ‘consolidation’ survey, designed to collect data that would allow the provisional staging framework to be refined. This second survey was improved for comprehensibility and presentation based on (i) published guidelines for online survey research design [14] and (ii) feedback from the exploratory survey. This survey was also hosted on the Opinio platform and was distributed via email to caregivers who were members of the UK PPA Support Group as well as PPA support groups in Melbourne and Sydney, Australia. Both current and bereaved caregivers were included in the survey to capture information about late‐stage disease. Data were collected between February 2020 and April 2020 for UK PPA Support Group respondents and between January 2021 and May 2021 for Australian support group respondents. As before, all caregivers had longstanding personal contact with the patient whose illness they described.

In the first section of the survey, caregiver respondents selected that the diagnosis of the person they were answering the survey about was lvPPA. They then provided information about their relationship to the patient, and the patient's age at the time of the survey, at symptom onset, when first medically assessed and when diagnosed.

In the second section, symptom lists under each stage were presented. The symptom labels presented here are given in full in Table S2. For each symptom, survey respondents were asked to indicate whether (based on proximity to the other symptoms and overarching stage descriptor; Table S1) the symptom began at the stage to which it had provisionally been assigned, if it began at an earlier or later stage (and, if so, which one) or if it was absent altogether. For each stage, participants were asked to indicate the overall duration of that stage. Recognizing that respondents with patients in earlier stages of illness might not recognize most symptoms assigned provisionally to later lvPPA stages, and to prevent potential distress by confronting respondents with unanticipated symptoms, respondents were permitted to discontinue this section of the survey at any point. The point at which the respondent chose to discontinue this section of the survey was considered indicative of the patient's current lvPPA stage. Participants were able to review and edit their responses at any point via a ‘Back’ button.

In the final section of the survey, respondents were presented with a representative list of symptoms present (1) in the other PPA variants on which there has been a previous publication [8] (sampling each of the domains A, B1 and B2) and (2) (mainly as an internal ‘control’ to assess response bias) in a staging system for a clinically distinct, ‘visuospatial’ dementia (posterior cortical atrophy) [12]: for each of these symptoms, caregivers were again asked to indicate whether the symptom was present and, if so, to which stage it should be assigned. Respondents were also given the opportunity to make additional comments about symptoms not covered elsewhere in the survey, and their impressions of the staging system in its current form, for the purpose of qualitative analysis.

### Quantitative analysis of survey responses

For each symptom in the consolidation survey, the percentage of respondents who had declared that symptom to be ‘present’, regardless of the stage at which it was endorsed, was calculated; a symptom was retained only if a majority (at least 50%) of caregivers who provided a response to a given symptom reported it was present at some stage. The percentage of respondents who judged that each symptom had been assigned to the correct stage was also calculated: if a majority considered that a symptom should be reassigned to an earlier or later stage, it was reassigned accordingly. If a symptom was jointly assigned to more than one stage (i.e., if the majority was tied across two or more stages), it was retained only at the earliest stage (because, generally, the earliest appearance of a symptom is most relevant for planning care needs and/or signalling disease progression). ‘Confidence’ of staging for each symptom was assessed as the proportion of respondents endorsing that symptom in its final stage assignment.

### Selection of functional ‘milestone’ symptoms

From the full list of symptoms that were retained for inclusion in the staging system, ‘milestone’ symptoms that were likely to signal significant illness transitions relevant to occupational and social activities, personal needs and other aspects of daily‐life functioning were identified.

### Qualitative analysis of survey responses

Caregiver comments on the exploratory and consolidation surveys were analysed qualitatively using framework analysis [15, 16]. A tentative framework was proposed by one of the authors (CJDH) after familiarization with a wider dataset, including qualitative responses to the surveys completed by caregivers for people with other PPA syndromes, described previously [8]. This initial coding framework was then applied to 20% of the dataset, which was then reviewed by another author (EH). Discrepancies or alternative interpretations were reviewed and discussed until a consensus was reached. Based on this consensus, a thematic framework was developed using tables of data in Microsoft Excel (v2016) and applied to the full lvPPA survey dataset.

### Ethical approval

Data collected from UK PPA Support Group members were collected under the Rare Dementia Support Impact Study, a protocol for which has been published separately [17]. Ethical approval was granted by the University College London Research Ethics Committee (8545/004: Rare Dementia Support [RDS] Impact Study). Additional local site approval for support group members in Sydney was granted by the South Eastern Sydney Local Health District HREC (2020/ETH02530). All respondents gave informed consent, following Declaration of Helsinki guidelines.

### Data sharing

The data that support the findings of this study are available on request from the corresponding author. The data are not publicly available as they include information that could compromise the privacy of the research participants.

## RESULTS

**FIGURE 1:**
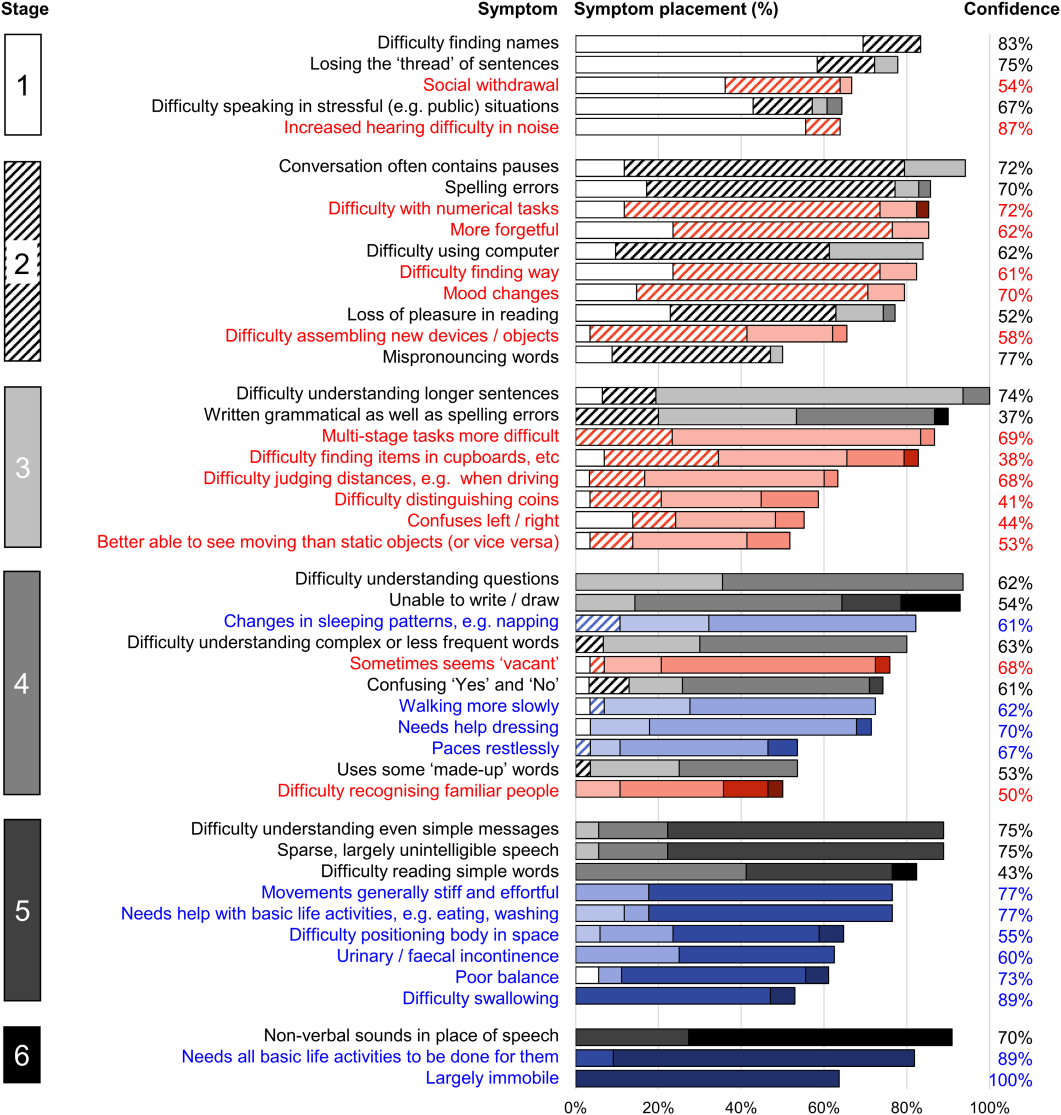
Symptom frequencies and confidence in symptom placement by clinical stage for logopenic variant primary progressive aphasia. A symptom was retained only if a majority (at least 50%) of caregivers who provided a response to a given symptom reported it was present at some stage of the illness (see Appendix S1, Table S2, for complete symptom list). Boxes on the left‐hand side denote stages, numbered 1 (very mild) to 6 (profound). Written symptom labels and bars are colour‐coded based on domains of verbal communication (grey scale) and non‐verbal functioning (non‐verbal cognition and behaviour, red; personal care and well‐being, blue). Horizontal bars indicate the percentage of respondents to a given symptom who indicated that symptom to be ‘present’, with subdivisions of each bar reflecting the proportion of respondents indicating that symptom to be present at a specific stage. Percentages in the ‘confidence’ column were calculated as the percentage of people who had responded that a given symptom was present who endorsed placement of that symptom in its final stage (i.e., the highest agreement achieved for placement of that symptom); this varied across stages (stage 1 mean average, 73%; stage 2, 66%; stage 3, 53%; stage 4, 61%; stage 5, 69%; stage 6, 86%). Symptoms have been ordered within stages in descending order of overall frequency.

Demographic and clinical characteristics for the consolidation survey cohort are presented in Table 1; characteristics of the stages endorsed in the consolidation survey are presented in Table 2. The final stages are presented in Figure 1 and associated milestone symptoms in Table 3. Raw data supporting the stage assignments are shown in Table S2, and themes, subthemes and illustrative caregiver comments from the qualitative framework analysis in Table 4.

**TABLE 1:** Breakdown of demographic and clinical characteristics for the cohort included in the consolidation caregiver survey.

	*N*/mean (SD)
UK cohort (*n*)	27
Australian cohort (*n*)	10
All survey caregiver respondents (*n*)	37
Caregiver respondents for deceased patients	2
Relationship status (partner/other, *n*)	28/8^a^
Age at which first symptom noticed	63.41 (7.65)
Age at first GP appointment	64.72 (7.74)
Delay seeking medical advice (years)	1.31 (1.45)
Age at diagnosis	66.08 (7.63)
Time to diagnosis (years)	2.66 (1.89)
Age at survey	69.71 (6.73)^b^

*Note*: The table shows demographic and clinical characteristics for the patient cohort unless otherwise indicated. Mean (standard deviation) data are presented unless otherwise specified. Not all questions were answered by all respondents, and missing data are coded as follows: ^a^ *n*−1; ^b^ *n*−2.

Abbreviation: GP, general practitioner.

**TABLE 2:** Characteristics of the six stages endorsed by the cohort included in the consolidation caregiver survey.

Stage	*N* patients	*N* symptoms endorsed	Estimated stage duration (years)
Stage 1	1	5	2.26 (1.68) Range 6 months to 8 years *N* = 29
Stage 2	5	10	1.75 (1.10) Range 6 months to 4 years *N* = 21
Stage 3	4	8	1.63 (1.03) Range 4 months to 3.5 years *N* = 13
Stage 4	14	11	1.02 (0.53) Range 2 months to 1.5 years *N* = 7
Stage 5	4	9	1.33 (0.76) Range 6 months to 2 years *N* = 3
Stage 6	9	3	No data

*Note*: The table shows the characteristics of the six stages endorsed by the cohort included in the consolidation caregiver survey. The *N patients* column gives the number of patients estimated to be in that stage at the time of the survey or at death (see text for details). The *N symptoms endorsed* column gives the number of symptoms adopted under each stage by the cohort. The *Estimated stage duration* column only includes data for caregiver respondents for patients who had progressed to at least the next stage, that is, the estimated duration of stage 1 does not include an estimate for the person still in that stage. This question was not compulsory and several respondents felt unable to put a value on this.

**TABLE 3:** Clinical stages with milestone symptoms in logopenic variant primary progressive aphasia.

Stage	Functional milestone symptoms	Daily‐life implications	Care needs
1. Very mild	Communication Difficulty finding names Difficulty speaking in stressful situations Increased difficulty hearing in noise	Problem becomes noticeable to others May need to stop work (if depends strongly on verbal competence)	Speech and language therapy (communication strategies) Occupational and financial counselling Psychological support/counselling Support groups (combat isolation, share strategies)
2. Mild	Communication Mispronouncing words Other Difficulty with numerical tasks Difficulty using computer Difficulty finding way Difficulty assembling new devices/objects	Will generally need to stop work (a range of occupations potentially affected)	Speech and language therapy (communication strategies) Occupational therapy assessment in home environment Navigational aids Psychological support/counselling, support groups
3. Moderate	Communication Difficulty understanding longer sentences Other Difficulty finding items in cupboards etc. Difficulty judging distances, e.g. driving	Requires adapted communication Will often need to stop driving	Speech and language therapy (communication aids) Transport assistance Accessible (non‐verbal) social activities, e.g. art, music Supervision with medications and meal preparation Assistance with finances, other personal administrative tasks Psychological support/counselling, support groups
4. Severe	Communication Difficulty understanding questions Other Needs help dressing Difficulty recognizing familiar people	Care support will often be necessary	Part‐time carers and supervision Speech and language therapy (communication aids) Psychological support/counselling, support groups
5. Very severe	Communication Difficulty understanding simple messages Sparse, largely unintelligible speech Other Needs help with basic life activities, e.g. eating, washing Urinary/faecal incontinence Poor balance Difficulty swallowing	Residential care will often be necessary; marked restriction of activities and dependency in daily life Severe frailty	Full‐time carers and supervision Speech and language therapy (assessment of swallowing, nutrition, non‐verbal communication strategies) Occupational therapy and physiotherapy assessments, mobility aids Continence management Psychological support/counselling, support groups
6. Profound	Communication Mute except for non‐verbal sounds Other Needs all basic life activities undertaken on their behalf Largely immobile	Full dependency Very severe frailty	Complete nursing care with assessment of nutrition/indication for assisted feeding, complications of immobility etc. Psychological support/counselling, support groups

*Note*: The table identifies ‘milestone’ symptoms with significant implications for daily‐life functioning and care in logopenic variant primary progressive aphasia (lvPPA), and the clinical stages at which these first develop (described more fully in Figure 1). Respondent consensus on milestone symptom staging was high across stages, with the best‐performing milestone symptom (in terms of consensus as to this symptom being placed in the correct stage) as follows: stage 1, ‘Increased hearing difficulty in noise’, 87%; stage 2, ‘Mispronouncing words’, 77%; stage 3, ‘Difficulty understanding longer sentences’, 74%; stage 4, ‘Needs help dressing’, 70%; stage 5, ‘Difficulty swallowing’, 89%; stage 6, ‘Largely immobile’, 100%. Care needs (last column) will vary substantially between individuals, particularly in earlier stages, and subsume the needs of the individual affected by lvPPA, that individual's caregivers and their interaction; a number of the items listed are multi‐componential, with stage‐dependent emphasis (e.g., psychological support may entail adjustment to the diagnosis and loss of independence earlier on, and coping with social isolation, altered relationships and caregiver depression in later stage disease).

**TABLE 4:** Qualitative framework analysis: themes, subthemes and illustrative comments from caregivers of patients with logopenic variant primary progressive aphasia.

Theme/subtheme	Illustrative caregiver comments
Theme 1: Impact and experience of symptoms	
Emotional impact of the condition	‘She occasionally gets upset and tearful which is new for her. Even when she had cancer I never saw her upset or negative. She realizes this is not going to be pleasant and I think that worries her greatly. For me the frustration is that there is little I can do to slow this down’
Earliest symptoms noticed	‘I had noticed a struggle for words and some memory loss for a good 2/3 years leading up to diagnosis’
Adding additional information about symptoms already listed in the stages	‘Relatively, my person is still physically well. Sometimes I think, because she can't see very well, that slows her down and prevents falls for instance but makes toileting problematic at times. All her movements are slower and it's hard to keep her moving’
Adding descriptions of symptoms not included	‘She has a voracious appetite and has since about stage 2’
Theme 2: Illness progression/trajectory	
Fluctuations in decline	‘In our case there have been quite long periods of stability, then a sudden worsening. Sometimes this seems to have been brought about by a change to routine or an outside event, a domestic problem (something breaking down), staying away from home even for a short period. The condition then settles down again but doesn't return to what it was before’
Speed of progression	‘It definitely seems like the first couple of years are gradual—and it is hard to notice changes until you look back after a year and realize they can't do that anymore, or have been struggling—like driving. Infections/delirium can massively exacerbate the illness and speed it up’
Theme 3: Experience of doing the research	
Difficulties answering questions on behalf of the patient	‘I feel I haven't been much help. In hindsight, many personality things of Mum might have been a sign years and years ago. Or they might have been just the way she is. Many things listed she is a million miles away from experiencing, whilst others listed affect her all day every day’
Difficulties with the way the survey was designed	‘I found it very difficult to answer many questions, as the descriptions given of the proposed stages rarely corresponded to the actual progression of my partner (or of other friends living with PPA) and seemed to be based on pre‐existing assumptions about the course and symptoms of the disease. Has any thought been given to using free descriptive text? I could more easily write a descriptive, chronological narrative. I really question the methodology used here’
Theme 4: Utility of the stages	
Perceived strengths of the stages	‘I do thank you very very much for compiling the stages. It really does help to give an understanding of PPA and the future and also increases my patience when I realize it is condition related and not just her being irritating. Haha’
Perceived limitations of the stages	‘Regarding staging, the difficulty is like trying to decide where the boundaries lie between yellow, orange and red in the rainbow—making sharp boundaries between items on a continuous “spectrum” can only be approximate. But I understand the need to try!’
Theme 5: Suggestions for further development/dissemination	
Incorporating care milestones/appropriate therapies into the stages	‘Reference to types of therapies that may be helpful at later stages—input from neuro physios and neuro occupational therapists so that appropriate physical and other sensory therapies can be used when other activities become too difficult or do not maintain interest. Thank you for doing this’
Aligning stages with intact abilities	‘I think this is great but maybe would be also useful to add what the person IS still able to do as well as CAN'T’
Acknowledging individual differences	‘Thank you for doing this work. It feels like there will be a lot of variation from person to person—and their circumstances. For example as my mother was initially living alone issues with planning executive function may have come earlier compared to if my father had still been alive as I think he would have without even noticing taken some of this on and therefore ‘covered‐up’ these difficulties’

*Note*: The table presents themes and subthemes identified in the qualitative framework analysis, with illustrative quotations representing each subtheme from caregivers of people living with logopenic variant primary progressive aphasia.

Abbreviation: PPA, primary progressive aphasia.

Mean age of symptom onset for patients comprising the consolidation survey cohort was 63.4 (standard deviation 7.65) years and mean delay to diagnosis 2.66 (1.89) years.

### Symptom‐based stages and functional milestones

The six‐stage framework (Figure 1) was endorsed by the consolidation survey cohort. As anticipated, symptoms relating to communication were present at stage 1; earliest symptoms included difficulty conversing in stressful situations and recalling names, and a tendency to lose the thread of what one was saying, as well as non‐verbal symptoms relevant to communication function, notably increased difficulty hearing in noise and social withdrawal. Additionally, other non‐verbal symptoms relating to episodic and topographical memory, numeracy, praxis and mood were endorsed as early as stage 2. Difficulties with visual perception were endorsed from stage 3, and increased dependency with personal care (e.g., needing assistance with dressing) from stage 4. Respondent consensus on symptom staging was good for early and late stages but reduced at intermediate stages (stage 1, mean average 73%; stage 3, 53%; stage 6, 86%) (Figure 1). Survey respondents were also asked to estimate the duration of each stage; means, standard deviations and ranges for each stage are presented in Table 2.

For each stage, milestone symptoms with significant implications for daily‐life functional transitions and care needs were identified (Table 3). Milestones were linked to communication and (from stage 2) non‐verbal functions; sequentially, these are likely to impact ability to work (stages 1 and 2), live independently (stages 3 and 4) and maintain quality of life with severe cognitive and neurological disability (stages 5 and 6). Additional specific language symptoms may help clinicians assign a stage in suspected lvPPA (Figure 1): these include relatively isolated word‐finding difficulty (stage 1), spelling errors (stage 2), grammatical errors (stage 3), difficulty understanding questions (stage 4) and unintelligible speech (stage 5).

### Comparison with other PPA syndromes

Increased difficulties with hearing in noise, memory and navigation developed earlier and problems with mobility and sleep began later in lvPPA than previously reported in other PPA syndromes [8]; spelling errors were an early feature across syndromes, whilst loss of meaningful communication and impaired walking, self‐care, swallowing and continence were late‐stage features across all PPA syndromes.

Of the 15 additional ‘control’ symptoms relevant to posterior cortical atrophy presented in the survey, six (reflecting non‐verbal parietal lobe functions, i.e., relating to praxis and visuoperceptual awareness) were endorsed by caregivers for inclusion in the stages for lvPPA (Table S2), compared with two for semantic variant PPA (svPPA) and four for nonfluent variant PPA (nfvPPA) [8].

Spotlighted a subtheme, ‘Acknowledging individual differences’, that was identified by caregivers for patients with lvPPA but not other PPA syndromes.

### Qualitative analysis of survey responses

The qualitative framework comprised five major themes identified in our previous analysis of nfvPPA and svPPA qualitative responses [3] (Table 4): (i) impact and experience of symptoms; (ii) illness progression/trajectory; (iii) experience of doing the research; (iv) utility of the stages; (v) suggestions for future development/dissemination. Thirteen subthemes were identified within these major themes, and together themes and subthemes encompassed respondents' experiences of living with lvPPA and of the staging survey, and their suggestions for further development/dissemination. One subtheme under ‘Suggestions for further development/dissemination’ was identified for lvPPA that was not present for nfvPPA/svPPA (‘Acknowledging individual differences’) and one subtheme identified for nfvPPA/svPPA was not identified for lvPPA (‘Importance of how and when information is accessed’).

## DISCUSSION

Here a six‐stage scheme and candidate milestones for signposting symptom onset and functional progression in lvPPA have been presented, based on the lived experience of caregivers. Early symptoms included problems with hearing in noise, situational word‐finding difficulty with loss of message comprehension and speech intelligibility signalling later‐stage progression. Additionally, problems with memory and route‐finding were prominent early non‐verbal symptoms, and (as in other PPA syndromes [8]) late‐stage disease was characterized by generalized impairments of communication, cognition, mobility and self‐care, leading to full functional dependence. The lengthy mean diagnostic delay (2.66 years) underscores the need for new clinical markers of lvPPA.

Neurobiologically, the symptom sequence identified here fits with the known spread of AD pathology through temporo‐parietal cortices [18], overlapping phenotypically with posterior cortical atrophy (the ‘visual variant’ of AD) and typical memory‐led AD [19, 20]. Our findings corroborate recent formulations of lvPPA as a multidimensional AD phenotype within the wider syndromic spectrum of AD, grounded in shared neural network anatomy [19, 20, 21, 22]. Syndromic phenotypes converge with disease progression: for example, a language profile similar to lvPPA develops in posterior cortical atrophy [2], the AD‐linked communication phenotype evolving at different rates across syndromes.

This work highlights the phenotypic breadth of lvPPA: certain features, such as early impairment of hearing in noise, are highly relevant to daily‐life communication but not part of standard assessments of language function in AD, or indeed current consensus diagnostic criteria for lvPPA [4]. These findings corroborate previous findings of a complex auditory phenotype in lvPPA that encompasses impaired phonemic processing [23, 24] and dichotic listening [25], with deficits in the disambiguation of foreground sounds (e.g., speech) from background noise now having been identified across the AD spectrum [26, 27]. In patients with these difficulties, an important clinical implication is that hearing aids that simply boost the incoming signal are likely to have limited benefit for everyday communicative listening [28].

Further, communication and other functional milestones in lvPPA are likely to reflect complex interactions between language impairment and amnestic, visuospatial and motor deficits. The lower ‘confidence’ in symptom placement for mid‐stage compared with early‐ and late‐stage lvPPA (Figure 1) accords with the individual clinical variability highlighted by the qualitative analysis and by previous work in lvPPA [6, 19, 20]. This phenotypic diversity underlines the need to stage personalized illness trajectories in lvPPA, both for early consideration of disease‐modifying therapies [9] and for accessing appropriate non‐pharmacological interventions (such as speech and language therapy) and support throughout the illness (Table 3).

A number of candidate scoring instruments are currently available for lvPPA, including the Progressive Aphasia Severity Scale [29], Mini Linguistic State Examination [30], Frontotemporal Dementia Rating Scale [13] and Clinical Dementia Rating Scale plus National Alzheimer's Coordinating Center Frontotemporal Lobar Degeneration module [31]. Prospective validation will be required to fully assess the place of the new system proposed here in relation to the existing instruments; however, it is felt that there are two key ways in which the work presented here will add value. First, scales are typically and inherently reductionist, designed to give a brief snapshot of where an individual is in their illness; the symptoms presented in Figure 1 are highly granular, providing a detailed roadmap of the illness (and individual trajectories through the illness). Secondly, the staging proposed here foregrounds communication functions that are not emphasized by other scales but which are of paramount importance in a ‘language‐led’ dementia. The complexity and variability of the symptoms arrayed in Figure 1 underline that staging the individual person with lvPPA should only be undertaken as part of a consultation between patient, caregiver and clinician—so that the stage can be interpreted and management tailored according to their personal circumstances.

This study has limitations that should direct future work. Caregiver reports were retrospective and possibly subject to recall bias; prospective, longitudinal studies are required. This is illustrated most tellingly in the data on stage durations presented in Table 2: the overall range for some individual stages was extremely wide (e.g., stage 1 ranged from 6 months to 8 years), and several caregivers felt unable to estimate stage durations, speaking to the inherent difficulty in applying, retrospectively, a categorical distinction onto a continuous process. The symptom list presented to caregiver respondents in the consolidation survey was heavily weighted toward cognitive and functional symptoms of lvPPA, and so did not fully cover neuropsychiatric symptoms that are likely to hold significant clinical relevance for patients and caregivers. This raises the broader issue of the weighting of symptoms used to define particular stages or milestones—different symptoms are not functionally equivalent and their impact is likely to vary as the disease evolves. The future clinical application of the staging system will require this weighting to be defined prospectively. Our proposed milestone symptoms also need to be prospectively validated against measures of daily‐life impact. Furthermore, detailed information was not collected on the pathways to diagnosis and biomarkers were not available to corroborate the clinical syndromic diagnosis. Whilst the findings are interpreted here as having potential relevance to the wider AD spectrum, it should be acknowledged that other pathologies have been associated with the lvPPA phenotype [32, 33, 34] and so the specificity of this work to AD more broadly is yet to be established. The list of symptoms presented to respondents here was developed initially with UK‐based, English‐speaking caregivers in mind: it is unlikely that an identical symptom ordering or even the same set of symptoms will apply to lvPPA developing in speakers of other languages. Future work should engage larger, more socio‐culturally and linguistically diverse cohorts, representing all major AD variant syndromes, ideally with biomarker correlation. Individuals will move through the stages at different rates, and this requires definition. Head‐to‐head comparisons with existing AD severity scales [7] are needed, to develop clinical scales and care pathways for anticipating and managing communication dysfunction across the AD spectrum.

## AUTHOR CONTRIBUTIONS

**Chris J. D. Hardy:** Conceptualization; investigation; funding acquisition; writing – original draft; methodology; visualization; formal analysis; data curation. **Cathleen Taylor‐Rubin:** Investigation; writing – review and editing. **Beatrice Taylor:** Writing – review and editing; data curation. **Emma Harding:** Methodology; writing – review and editing; data curation. **Aida Suarez Gonzalez:** Writing – review and editing. **Jessica Jiang:** Writing – review and editing. **Laura Thompson:** Writing – review and editing; investigation. **Rachel Kingma:** Writing – review and editing; investigation. **Anthipa Chokesuwattanaskul:** Writing – review and editing. **Ffion Walker:** Writing – review and editing; investigation. **Suzie Barker:** Writing – review and editing; project administration. **Emilie Brotherhood:** Writing – review and editing; project administration. **Claire Waddington:** Writing – review and editing. **Olivia Wood:** Writing – review and editing. **Nikki Zimmermann:** Writing – review and editing. **Nuriye Kupeli:** Writing – review and editing. **Keir X. X. Yong:** Writing – review and editing. **Paul M. Camic:** Writing – review and editing. **Joshua Stott:** Writing – review and editing. **Charles R. Marshall:** Writing – review and editing. **Neil P. Oxtoby:** Writing – review and editing. **Jonathan D. Rohrer:** Writing – review and editing. **Frankie O'Shea:** Writing – review and editing. **Anna Volkmer:** Writing – review and editing; resources. **Sebastian J. Crutch:** Writing – review and editing; funding acquisition; conceptualization; supervision. **Jason D. Warren:** Conceptualization; funding acquisition; writing – review and editing; methodology; formal analysis; data curation; supervision; resources.

## FUNDING INFORMATION

The Dementia Research Centre is supported by Alzheimer's Research UK, Brain Research UK and the Wolfson Foundation. This work was supported by the Alzheimer's Society, the Royal National Institute for Deaf People, the National Institute for Health Research University College London Hospitals Biomedical Research Centre, the University College London Leonard Wolfson Experimental Neurology Centre (grant PR/ylr/18575) and the Rare Dementia Support Impact Study (funded by the Economic and Social Research Council and National Institute for Health Research; funding reference ES/S010467/1). CJDH was supported by a Royal National Institute for Deaf People—Dunhill Medical Trust Pauline Ashley Fellowship (grant PA23_Hardy), a Wellcome Trust Institutional Strategic Support Fund award (204841/Z/16/Z) and an NIHR Grant (NIHR204280). EH was funded by an Economic and Social Research Council Postdoctoral Fellowship award (ES/W006014/1). ASG received grant support from ESRC‐UKRI (ES/Y007484/1) and NIHR (COV‐LT2‐0014 and NIHR203680). JJ was supported by a Frontotemporal Dementia Research Studentship in Memory of David Blechner (funded through the National Brain Appeal). NZ and the RDS PPA Support Group are supported by the National Brain Appeal. NK is supported by Alzheimer's Society Junior Fellowship grant funding (Grant Award number 399 AS‐JF‐17b‐016). KY is an Etherington PCA Senior Research Fellow and is funded by the Alzheimer's Society, grant number 453 (AS‐JF‐18‐003), and a USA NIH grant R01EY027964. JS is funded by the NIHR (NIHR programme grant for applied research, NIHR203680). CRM was supported by an NIHR Grant (NIHR204280). AV is funded by an NIHR Advanced Fellowship (NIHR302240). JDW received grant support from the Alzheimer's Society, Royal National Institute for Deaf People and the National Institute for Health Research University College London Hospitals Biomedical Research Centre. NPO is a UKRI Future Leaders Fellow (MR/S03546X/1). BT is supported by a UCL, Bloomsbury and East London ESRC Doctoral Training Partnership (ES/P000592/1). This study is funded by the NIHR (Invention for Innovation, NIHR204280). The views expressed are those of the authors and not necessarily those of the NIHR or the Department of Health and Social Care. This research was funded in whole, or in part, by the Wellcome Trust (204841/Z/16/Z).

## CONFLICT OF INTEREST STATEMENT

JDR has provided consultancy or served on the medical advisory board for Prevail, Novartis, Wave Life Sciences, Alector, Aviado Bio, Arkuda Therapeutics and Denali Therapeutics.

## Supporting information


Appendix S1

## Data Availability

The data that support the findings of this study are available from the corresponding author upon reasonable request.
