# Supplementary material for: Symptom‐based staging for logopenic variant primary progressive aphasia
Source: Eur J Neurol. 2024 Apr 26;31(7):e16304. doi: 10.1111/ene.16304 (PMC11235891; doi:10.1111/ene.16304)
Supplement: Supplementary file 1 — Appendix S1 [file ENE-31-e16304-s001.docx]

**SUPPLEMENTARY MATERIAL.** Symptom-based staging for logopenic variant primary progressive aphasia, by CJD Hardy et al.

**Supplementary Table S1.** Descriptions of stages presented to caregivers in the consolidation survey

| **PPA stage** | **Daily life impact** |
| --- | --- |
| **1: Very mild** | This stage reflects the earliest clinical manifestations of PPA, but the person may not seek help, attributing problems to stress or ageing. Symptoms may be intermittent, difficult for the person to describe, or recognised only by those who know them well or in hindsight. |
| **2: Mild** | Communication and other less prominent problems with everyday activities are generally evident to others as well as to the person themselves (though sometimes insight may be lacking). |
| **3: Moderate** | The person may now require help managing certain aspects of day-to-day life and will generally have to stop working. Communication difficulties tend to frustrate important goals and social activities. |
| **4: Severe** | The person now requires support with many aspects of daily living and communication is increasingly difficult. They may no longer be able to live independently. |
| **5: Very severe** | Cognitive and behavioural changes are more global in nature, and many are common to all PPA syndromes. Meaningful communication is rarely possible. The person is likely to need help with daily personal care including toileting, and physical symptoms will have developed to the extent that mobility is significantly affected. |
| **6: Profound** | Communication is now no longer possible. The person may lose their ability to respond to their environment and becomes largely immobile |

The Table shows cross-syndromic stage labels that were presented to survey respondents in the present study, and our previous publication of symptom-led staging in the nonfluent/agrammatic and semantic variants of primary progressive aphasia^1^. The stage labels align with those used in the Frontotemporal Dementia Rating Scale^2^. The text in the ‘Daily life impact’ column was developed during exploratory work and adapted from descriptors used in posterior cortical atrophy^3^ and Alzheimer’s disease^4^, and intended to help caregiver survey respondents as a symptom ordering tool.

**Supplementary Table S2.** Detail of survey items and responses from caregivers for people with logopenic variant primary progressive aphasia

| **STAGE 1: Very Mild** | | | | | | | | | | | | |
| --- | --- | --- | --- | --- | --- | --- | --- | --- | --- | --- | --- | --- |
| **Original symptom descriptor** | **N responses** | **% present** | **% correct [S1]** | **% S2** | **%S3** | **%S4** | **%S5** | **%S6** | **Action** | **Succinct item wording** | **Neurological interpretation** |  |
| Particular problems hearing in busy environments, e.g. a noisy room or a dinner party. | 36 | 63.9 | **87.0** | 13.0 | 0.0 | 0.0 | 0.0 | 0.0 |  | Increased hearing difficulty in noise | Central auditory dysfunction |  |
| Difficulties finding names for people and things, though it feels to the person as if it’s on the ‘tip of their tongue’. | 36 | 83.3 | **83.3** | 16.7 | 0.0 | 0.0 | 0.0 | 0.0 |  | Difficulty finding names | Anomia |  |
| Losing the ‘thread’ of sentences, e.g. starting to stay something but then trailing off. | 36 | 77.8 | **75.0** | 17.9 | 7.1 | 0.0 | 0.0 | 0.0 |  | Losing the 'thread' of sentences | Impaired verbal short term memory |  |
| *Particular problems when speaking in stressful situations, e.g. at a meeting or giving a presentation | 28 | 64.3 | **66.7** | 22.2 | 5.6 | 5.6 | 0.0 | 0.0 | Added to S1 | Difficulty speaking in stressful (e.g. public) situations | Impaired discourse |  |
| Becoming more withdrawn, not engaging with other people. | 36 | 66.7 | **54.2** | 41.7 | 4.2 | 0.0 | 0.0 | 0.0 |  | Social withdrawal | Socio-emotional dysfunction |  |
| **STAGE 2: Mild** | | | | | | | | | | | | |
| **Original symptom descriptor** | **N responses** | **% present** | **%S1** | **% correct [S2]** | **%S3** | **%S4** | **%S5** | **%S6** | **Action** | **Succinct item wording** | **Neurological interpretation** |  |
| Getting parts of words mixed up or confused with other words, e.g. saying “staffolding” instead of “scaffolding”, or “aminal” instead of “animal”. | 34 | 50.0 | 17.7 | **76.5** | 5.9 | 0.0 | 0.0 | 0.0 |  | Mispronouncing words | Phonological errors |  |
| Problems with confusion around numbers, e.g. struggling to work out how to split a bill in a restaurant, or how much change they should be owed. | 34 | 85.3 | 13.8 | **72.4** | 10.3 | 0.0 | 0.0 | 3.5 |  | Difficulty with numerical tasks | Dominant parietal dysfunction |  |
| Speech often contains pauses as the person searches for the right word to say. | 34 | 94.1 | 12.5 | **71.9** | 15.6 | 0.0 | 0.0 | 0.0 |  | Conversation often contains pauses | Anomia |  |
| Mood changes, such as becoming more irritable or anxious. | 34 | 79.4 | 18.5 | **70.4** | 11.1 | 0.0 | 0.0 | 0.0 |  | Mood changes | Affective alterations |  |
| Finding writing more difficult. Friends and family might start to notice spelling mistakes in emails and notes. | 35 | 85.7 | 20.0 | **70.0** | 6.7 | 3.3 | 0.0 | 0.0 |  | Spelling errors | Dysgraphia |  |
| Some problems with their memory for places or events | 34 | 85.3 | 27.6 | **62.1** | 10.3 | 0.0 | 0.0 | 0.0 |  | More forgetful | Impaired episodic memory |  |
| †Problems using a computer, e.g being slower to type or enter information | 31 | 83.9 | 11.5 | **61.5** | 26.9 | 0.0 | 0.0 | 0.0 | Added to S2 | Difficulty using computer | Activities of daily living |  |
| Finding their way, especially in new places, more difficult. | 34 | 82.4 | 28.6 | **60.7** | 10.7 | 0.0 | 0.0 | 0.0 |  | Difficulty finding way | Topographical agnosia |  |
| †Difficulty assembling new devices/objects | 29 | 65.5 | 5.3 | **57.9** | 31.6 | 5.3 | 0.0 | 0.0 | Added to S2 | Difficulty assembling new devices/objects | Apraxia |  |
| Loss of pleasure in reading, e.g. somebody who was previously a big reader may start reading less than they did before. | 35 | 77.1 | 29.6 | **51.9** | 14.8 | 3.7 | 0.0 | 0.0 |  | Loss of pleasure in reading | Dominant parietal dysfunction |  |
| **STAGE 3: Moderate** | | | | | | | | | | | | |
| **Original symptom descriptor** | **N responses** | **% present** | **%S1** | **%S2** | **% correct [S3]** | **%S4** | **%S5** | **%S6** | **Action** | **Succinct item wording** | **Neurological interpretation** |  |
| Problems understanding complicated or long sentences. | 31 | 100.0 | 6.5 | 12.9 | **74.2** | 6.5 | 0.0 | 0.0 |  | Difficulty understanding longer sentences | Receptive agrammatism |  |
| Complicated tasks with multiple steps that the person previously found easy (e.g. cooking an elaborate meal) become more difficult and take the person longer to complete. | 30 | 86.7 | 0.0 | 26.9 | **69.2** | 3.9 | 0.0 | 0.0 |  | Multi-stage tasks more difficult | Executive dysfunction |  |
| Problems with ‘spatial orientation’ emerge – the person may have difficulties judging distances or locating objects. | 29 | 65.5 | 5.3 | 21.1 | **68.4** | 5.3 | 0.0 | 0.0 |  | Difficulty judging distances, e.g. when driving | Visuospatial dysfunction |  |
| †Being able to see some things but not others, for instance following things that move (e.g. a ball being thrown) but not being able to find static objects (e.g. a ball lying still on the grass in the garden). | 29 | 51.7 | 6.7 | 20.0 | **53.3** | 20.0 | 0.0 | 0.0 | Added to S3 | Better able to see moving than static objects (or vice versa) | Visuospatial dysfunction |  |
| †Mixing up left and right | 29 | 55.2 | 25.0 | 18.8 | **43.8** | 12.5 | 0.0 | 0.0 | Added to S3 | Confuses left / right | Dominant parietal dysfunction |  |
| †Become confused when handling coins due to difficulties telling them apart | 29 | 58.6 | 5.9 | 29.4 | **41.2** | 23.5 | 0.0 | 0.0 | Added to S3 | Difficulty distinguishing coins | Dominant parietal dysfunction |  |
| †Difficulty finding things in a handbag, cupboard, etc. | 29 | 82.8 | 8.3 | 33.3 | **37.5** | 16.7 | 4.2 | 0.0 | Added to S3 | Difficulty finding items in cupboards, etc | Visuospatial dysfunction |  |
| The person makes grammatical as well as spelling errors in emails and notes, etc. | 30 | 90.0 | 0.0 | 22.2 | **37.0** | 37.0 | 0.0 | 3.7 | Moved to S3 from S4 | Written grammatical as well as spelling errors | Dysgraphia, expressive agrammatism |  |
| Repetitive/obsessive behaviours | 31 | 48.4 | 13.3 | 6.7 | 60.0 | 20.0 | 0.0 | 0.0 | Removed - not above 50% threshold |  | Obsessionality |  |
| **STAGE 4: Severe** | | | | | | | | | | | | |
| **Original symptom descriptor** | **N responses** | **% present** | **%S1** | **%S2** | **%S3** | **% correct [S4]** | **%S5** | **%S6** | **Action** | **Succinct item wording** | **Neurological interpretation** |  |
| Able to carry out some elements of personal care satisfactorily, but needing assistance with some things, e.g. dressing, shaving. | 28 | 71.4 | 5.0 | 0.0 | 20.0 | **70.0** | 5.0 | 0.0 |  | Needs help dressing | Activities of daily living |  |
| Appearing to be more vacant and not as alert as previously. | 29 | 75.9 | 4.6 | 4.6 | 18.2 | **68.2** | 4.6 | 0.0 |  | Sometimes seems 'vacant' | Alertness |  |
| Pacing up and down/ constantly walking. | 28 | 53.6 | 0.0 | 6.7 | 13.3 | **66.7** | 13.3 | 0.0 |  | Paces restlessly | Akathisia |  |
| Problems understanding the meaning of more complex or less frequent words | 30 | 80.0 | 0.0 | 8.3 | 29.2 | **62.5** | 0.0 | 0.0 |  | Difficulty understanding complex or less frequent words | Verbal agnosia |  |
| Difficulties with questions, e.g. understanding when choices are being given. | 31 | 93.5 | 0.0 | 0.0 | 37.9 | **62.1** | 0.0 | 0.0 |  | Difficulty understanding questions | Receptive agrammatism |  |
| Moving more slowly than before, e.g. when walking from room to room, or getting up from a chair. | 29 | 72.4 | 4.8 | 4.8 | 28.6 | **61.9** | 0.0 | 0.0 |  | Walking more slowly | Parkinsonism |  |
| Changes in sleeping patterns, e.g. seeming more tired and napping during the day. | 28 | 82.1 | 0.0 | 13.0 | 26.1 | **60.9** | 0.0 | 0.0 |  | Changes in sleeping patterns, e.g. napping | Disordered sleep |  |
| Getting Yes and No mixed up, for instance saying “No” when asked if they would like a cup of coffee, but really meaning “Yes”. | 31 | 74.2 | 4.4 | 13.0 | 17.4 | **60.9** | 4.4 | 0.0 |  | Confusing 'Yes' and 'No' | Binary reversals |  |
| The person can no longer write or draw. | 14 | 92.9 | 0.0 | 0.0 | 15.4 | **53.9** | 15.4 | 15.4 | Moved to S4 from S6 | Unable to write / draw | Apraxia |  |
| Using made-up words that don’t exist, without seeming to be aware that what they have said doesn’t make sense. | 28 | 53.6 | 0.0 | 6.7 | 40.0 | **53.3** | 0.0 | 0.0 |  | Uses some 'made-up' words | Neologisms |  |
| *Problems recognising people. | 28 | 50.0 | 0.0 | 0.0 | 21.4 | **50.0** | 21.4 | 7.1 | Added to S4 | Difficulty recognising familiar people | Prosopagnosia |  |
| Needing reminding/ encouraging to use the toilet. | 28 | 39.3 | 0.0 | 0.0 | 9.1 | 81.8 | 9.1 | 0.0 | Removed - not above 50% threshold |  | Bladder / bowel dysregulation |  |
| **STAGE 5: Very severe** | | | | | | | | | | | | |
| **Original symptom descriptor** | **N responses** | **% present** | **%S1** | **%S2** | **%S3** | **%S4** | **% correct [S5]** | **%S6** | **Action** | **Succinct item wording** | **Neurological interpretation** |  |
| Problems with swallowing | 17 | 52.9 | 0.0 | 0.0 | 0.0 | 0.0 | **88.9** | 11.1 |  | Difficulty swallowing | Dysphagia |  |
| Requiring substantial help with many basic activities, e.g. eating and washing. | 17 | 76.5 | 0.0 | 0.0 | 15.4 | 7.7 | **76.9** | 0.0 |  | Needs help with basic life activities, e.g. eating, washing | Activities of daily living |  |
| Movements become slower and stiffer. | 17 | 76.5 | 0.0 | 0.0 | 0.0 | 23.1 | **76.9** | 0.0 |  | Movements generally stiff and effortful | Parkinsonism |  |
| Speech is sparse and largely unintelligible, and now limited to a few words or sounds that may not make sense. | 18 | 88.9 | 0.0 | 0.0 | 6.3 | 18.8 | **75.0** | 0.0 |  | Sparse, largely unintelligible speech | Mutism |  |
| Difficulties understanding all but the simplest messages. | 18 | 88.9 | 0.0 | 0.0 | 6.3 | 18.8 | **75.0** | 0.0 |  | Difficulty understanding even simple messages | Receptive agrammatism |  |
| Feeling unsteady and/or falling | 18 | 61.1 | 9.1 | 0.0 | 0.0 | 9.1 | **72.7** | 9.1 |  | Poor balance | Postural instability |  |
| Increasingly frequent trouble controlling their bladder or bowels. | 16 | 62.5 | 0.0 | 0.0 | 0.0 | 40.0 | **60.0** | 0.0 |  | Urinary / faecal incontinence | Bladder / bowel dysregulation |  |
| Difficulties with positioning the body in space, e.g. the person may seem to find it hard to work out how to position their body to get into a car. | 17 | 64.7 | 0.0 | 0.0 | 9.1 | 27.3 | **54.5** | 9.1 |  | Difficulty positioning body in space | Visuospatial dysfunction |  |
| Able to read and understand only some if any simple words | 17 | 82.4 | 0.0 | 0.0 | 0.0 | 50.0 | **42.9** | 7.1 |  | Difficulty reading simple words | Alexia |  |
| **STAGE 6: Profound** | | | | | | | | | | | | |
| **Original symptom descriptor** | **N responses** | **% present** | **%S1** | **%S2** | **%S3** | **%S4** | **%S5** | **% correct [S6]** | **Action** | **Succinct item wording** | **Neurological interpretation** |  |
| Unable to control most movements without extreme difficulty. The person is mostly confined to chair or bed. | 11 | 63.6 | 0.0 | 0.0 | 0.0 | 0.0 | 0.0 | **100.0** |  | Largely immobile | Parkinsonism |  |
| Unable to perform any acts of daily living and needing to be washed, dressed, fed, etc. by another person. | 11 | 81.8 | 0.0 | 0.0 | 0.0 | 0.0 | 11.1 | **88.9** |  | Needs all basic life activities to be done for them | Activities of daily living |  |
| There is now almost no speech at all, but the person may make strange sounds (e.g. teeth grinding / popping noises) or laughing, sometimes inappropriately. | 11 | 90.9 | 0.0 | 0.0 | 0.0 | 0.0 | 30.0 | **70.0** |  | Non-verbal sounds in place of speech | Mutism |  |
| **Other PPA items** | | | | | | | | | | | | |
| **Original symptom descriptor** | **N responses** | **% present** | **%S1** | **%S2** | **%S3** | **%S4** | **%S5** | **%S6** | **Action** | **Succinct item wording** | **Neurological interpretation** |  |
| Changes in food preferences or appetite. | 29 | 41.4 | 8.3 | 16.7 | 33.3 | 16.7 | 8.3 | 16.7 | Not added |  | Abnormal eating behaviour |  |
| Little insight into the fact that there is anything wrong. | 28 | 39.3 | 18.2 | 18.2 | 54.6 | 9.1 | 0.0 | 0.0 | Not added |  | Anosognosia |  |
| Hallucinations, e.g. seeing or hearing something that isn’t really there. | 28 | 39.3 | 0.0 | 18.2 | 36.4 | 27.3 | 18.2 | 0.0 | Not added |  | Hallucinations |  |
| Complaining about pains or feelings in the body that don’t seem to have an easy physical explanation, e.g. headaches, toothache, or pains and feelings in other body parts; and/ or feeling temperature differently to before. | 29 | 37.9 | 9.1 | 9.1 | 63.6 | 18.2 | 0.0 | 0.0 | Not added |  | Sensory dysregulation |  |
| Change in libido – an increase or decrease in sexual desire | 29 | 34.5 | 60.0 | 10.0 | 20.0 | 10.0 | 0.0 | 0.0 | Not added |  | Socio-emotional dysfunction |  |
| Problems recognising household items, e.g. attempting to use bleach as washing-up liquid. | 28 | 21.4 | 16.7 | 0.0 | 16.7 | 50.0 | 16.7 | 0.0 | Not added |  | Nonverbal agnosia |  |
| Starting to dislike certain music or sounds; and/ or complaining of tinnitus or a constant ringing in their ears. | 30 | 20.0 | 16.7 | 16.7 | 33.3 | 33.3 | 0.0 | 0.0 | Not added |  | Central auditory dysfunction |  |
| Showing a sudden love for a particular kind of music or band. | 28 | 14.3 | 25.0 | 0.0 | 0.0 | 25.0 | 50.0 | 0.0 | Not added |  | Central auditory dysfunction |  |
| **Items generally associated with posterior cortical atrophy** | | | | | | | | | | | | |
| **Original symptom descriptor** | **N responses** | **% present** | **%S1** | **%S2** | **%S3** | **%S4** | **%S5** | **%S6** | **Action** | **Succinct item wording** | **Neurological interpretation** |  |
| Difficulty detecting the edge of pavements, paths and steps etc | 29 | 44.8 | 0.0 | 0.0 | 23.1 | 46.2 | 23.1 | 7.7 | Not added |  | Visuospatial dysfunction |  |
| General ‘clumsiness’ (in people who were not previously clumsy), such as knocking things over (inaccurate reaching out), putting a glass down sideways, etc. | 28 | 42.9 | 16.7 | 0.0 | 16.7 | 33.3 | 16.7 | 16.7 | Not added |  | Apraxia, visuospatial dysfunction |  |
| Little jerky movements in the fingers, arms, or other parts of the body | 29 | 41.4 | 16.7 | 0.0 | 8.3 | 58.3 | 16.7 | 0.0 | Not added |  | Myoclonus |  |
| Experiencing vertigo or other balance problems | 29 | 31.0 | 11.1 | 11.1 | 22.2 | 33.3 | 22.2 | 0.0 | Not added |  | Balance |  |
| Difficulty using stairs because of problems with spatial judgment | 29 | 31.0 | 0.0 | 0.0 | 22.2 | 66.7 | 0.0 | 11.1 | Not added |  | Visuospatial dysfunction |  |
| Be ‘functionally blind’, requiring support in all visually-guided activities | 29 | 13.8 | 0.0 | 0.0 | 50.0 | 25.0 | 0.0 | 25.0 | Not added |  | Visuospatial dysfunction |  |
| Experiencing delusions, e.g. believing that their caregiver is an imposter | 29 | 13.8 | 0.0 | 25.0 | 25.0 | 25.0 | 25.0 | 0.0 | Not added |  | Delusions |  |
| Experiencing odd visual sensations (e.g. occasional changes or washes of colour in the centre or periphery of their vision) | 29 | 3.4 | 0.0 | 0.0 | 0.0 | 0.0 | 100.0 | 0.0 | Not added |  | Early visual dysfunction |  |
| Experiencing continued deterioration of sensory functions, e.g. partial or complete loss of response to touch | 29 | 0.0 | 0.0 | 0.0 | 0.0 | 0.0 | 0.0 | 0.0 | Not added |  | Sensory dysregulation |  |

The table summarises the detailed survey responses given by caregivers for people they cared for with logopenic variant primary progressive aphasia. If ≥ 50% of respondents indicated a particular symptom was present but of those, a majority indicated that it should have been assigned to an earlier/later stage (here numbered stage, S1 to 6), that symptom was reassigned accordingly for the final staging (see main text). Original symptom descriptor, the full wording for each symptom that was presented to respondents in the survey. N responses, total number of respondents for that symptom item; % present, percentage of respondents reporting that symptom as present in the person they care(d) for, at any stage of the disease; % earlier, percentage of respondents reporting that symptom occurred at an earlier stage than assigned; % correct, percentage of respondents reporting that symptom was assigned to the correct stage, referred to as ‘confidence’ in symptom placement in main manuscript and Figure 1; % later, percentage of respondents reporting that symptom occurred at a later stage. Action, summary of decision as to whether to include symptom in final staging system, and where (if cell is blank, this means the item was retained in the stage it was presented to respondents). Succinct item wording, reduction of original symptom descriptor, homogenised where possible across PPA syndromes. At the end of the survey, respondents were asked additional questions about symptoms typically present in other PPA syndromes (see Hardy et al., 2023) and in posterior cortical atrophy: here, respondents had to indicate whether the symptom was present/ absent, and if present, to assign that symptom to a specific stage. *Indicates that an item from another PPA syndrome was endorsed and incorporated into the relevant Stage; †indicates that a symptom often associated with posterior cortical atrophy was endorsed and incorporated into the relevant Stage.

**Supplementary references**

1. Hardy, C. J. D. *et al.* Symptom-led staging for semantic and non-fluent/agrammatic variants of primary progressive aphasia. *Alzheimer’s & Dementia* (2023) doi:10.1002/alz.13415.

2. Mioshi, E., Hsieh, S., Savage, S., Hornberger, M. & Hodges, J. R. Clinical staging and disease progression in frontotemporal dementia. *Neurology* **74**, 1591–1597 (2010).

3. Carton A, Henley S, Walton J, Crutch S. Posterior cortical atrophy: understanding and support. *The Journal of Dementia Care* **23**, 22–24 (2015).

4. Reisberg, B., Ferris, S. H., de Leon, M. J. & Crook, T. The Global Deterioration Scale for assessment of primary degenerative dementia. *Am J Psychiatry* **139**, 1136–1139 (1982).
